# Gastric Outlet Obstruction Secondary to Groove Pancreatitis Mimicking Pancreatic Cancer

**DOI:** 10.7759/cureus.77590

**Published:** 2025-01-17

**Authors:** Mena Louis, Nathaniel Grabill, Bolaji Ayinde, Brian Gibson

**Affiliations:** 1 General Surgery, Northeast Georgia Medical Center Gainesville, Gainesville, USA; 2 Surgery, Northeast Georgia Medical Center Gainesville, Gainesville, USA; 3 Internal Medicine, Northeast Georgia Medical Center Gainesville, Gainesville, USA; 4 Trauma and Acute Care Surgery, Northeast Georgia Medical Center Gainesville, Gainesville, USA

**Keywords:** alcoholic pancreatitis, chronic pancreatitis, gastric outlet obstruction, gastrojejunostomy, groove pancreatitis, nutritional support, pancreatic pseudocyst

## Abstract

Groove pancreatitis is a rare complication of chronic pancreatitis that can lead to severe manifestations, including gastric outlet obstruction. A 37-year-old man with a history of chronic alcohol use presented with recurrent epigastric pain radiating to the back, accompanied by nausea and vomiting. Imaging revealed features of chronic pancreatitis such as pancreatic calcifications, pseudocyst formation, and a mass-like lesion in the pancreatic head causing duodenal stenosis.

Despite medical management and endoscopic interventions like biliary and duodenal stenting, the patient experienced persistent symptoms due to complications such as stent migration and failure to relieve the obstruction. Nutritional support became essential because of malnutrition from exocrine insufficiency and the inability to tolerate oral intake. Differentiating between groove pancreatitis and pancreatic carcinoma was challenging, as imaging and clinical features overlapped, and repeated fine-needle aspirations were non-diagnostic.

Ultimately, due to near-complete duodenal obstruction unresponsive to endoscopic treatment, the patient underwent an open gastrojejunostomy. Postoperatively, he demonstrated significant improvement, with the resumption of oral intake and stabilization of his nutritional status. Management required a multidisciplinary approach, combining medical therapy, surgical intervention, nutritional support, and addressing alcohol dependence to prevent further pancreatic damage. Early recognition and appropriate treatment of groove pancreatitis and its complications are essential to improve outcomes and prevent disease progression.

## Introduction

Chronic pancreatitis is a progressive inflammatory disorder of the pancreas characterized by irreversible morphological changes and gradual loss of both exocrine and endocrine function [1]. Prolonged alcohol abuse is one of the most common etiologies, leading to fibrosis, calcification, and ductal obstruction within the pancreatic tissue. Patients often present with persistent abdominal pain, malabsorption, and weight loss, significantly affecting their quality of life [2].

Groove pancreatitis is a rare form of segmental chronic pancreatitis that specifically involves the anatomical "groove" between the pancreatic head, duodenum, and common bile duct (CBD) [3]. This condition poses a diagnostic challenge as it can closely mimic the clinical and radiological features of pancreatic head carcinoma [4]. Symptoms typically include severe abdominal pain, nausea, vomiting, and weight loss, with some cases progressing to gastric outlet obstruction (GOO) due to duodenal stenosis [5].

Groove pancreatitis specifically affects the anatomical area between the pancreatic head, the duodenum, and the CBD [5]. While some pancreatologists debate its classification as a subtype of chronic pancreatitis, identifying groove pancreatitis as a distinct disease pattern helps guide diagnosis and management due to its unique clinical and pathological features [6]. This condition often mimics pancreatic head carcinoma both clinically and radiologically, leading to significant diagnostic challenges [7]. Understanding the nuances of groove pancreatitis is essential for accurate diagnosis and appropriate management, especially since standard interventions like duodenal stenting or gastrojejunostomy are common and well-documented in the literature [7].

## Case presentation

A 37-year-old Hispanic man with a history of chronic alcohol use presented with a two- to three-month history of intermittent epigastric pain radiating to the back, exacerbated by food intake. Two days prior to admission, his pain intensified and became constant. He reported occasional use of ibuprofen over the past few weeks. Three weeks before admission, he experienced an episode of maroon-colored emesis and melena, but his hemoglobin was 14.1 g/dL (14-18 g/dL), and his fecal occult blood test was negative in the emergency department.

**Figure 1:**
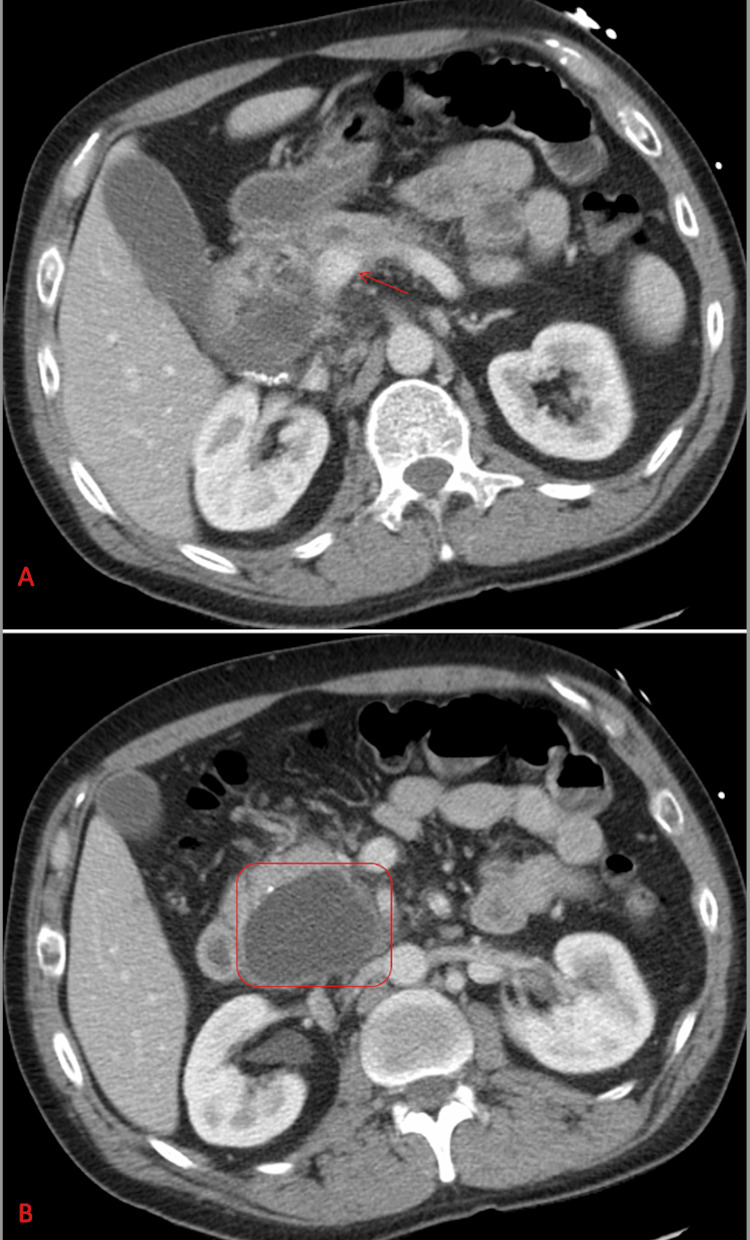
CT abdomen with IV contrast (axial view) showing peripancreatic stranding (red arrow, panel A), pancreatic calcifications, and a pancreatic pseudocyst measuring 6.2 × 4.1 cm (red box, panel B).

Laboratory studies revealed an elevated lipase level of 1,951 U/L (10-140 U/L) and an ethanol level of 98 mg/dL (0 mg/dL). Computed tomography (CT) of the abdomen showed peripancreatic stranding, pancreatic calcifications, and a pancreatic pseudocyst measuring 6.2 × 4.1 cm, increased from a previous size of 3.4 × 2.3 cm (Figure 1). The patient reported a prior episode of pancreatitis five years earlier and admitted to consuming three to four 16-ounce cans of beer daily. He was diagnosed with alcohol-induced pancreatitis and admitted for management. A right upper quadrant ultrasound was performed but did not reveal any gallstones. Instead, it showed sludge in the gallbladder.

**Figure 2:**
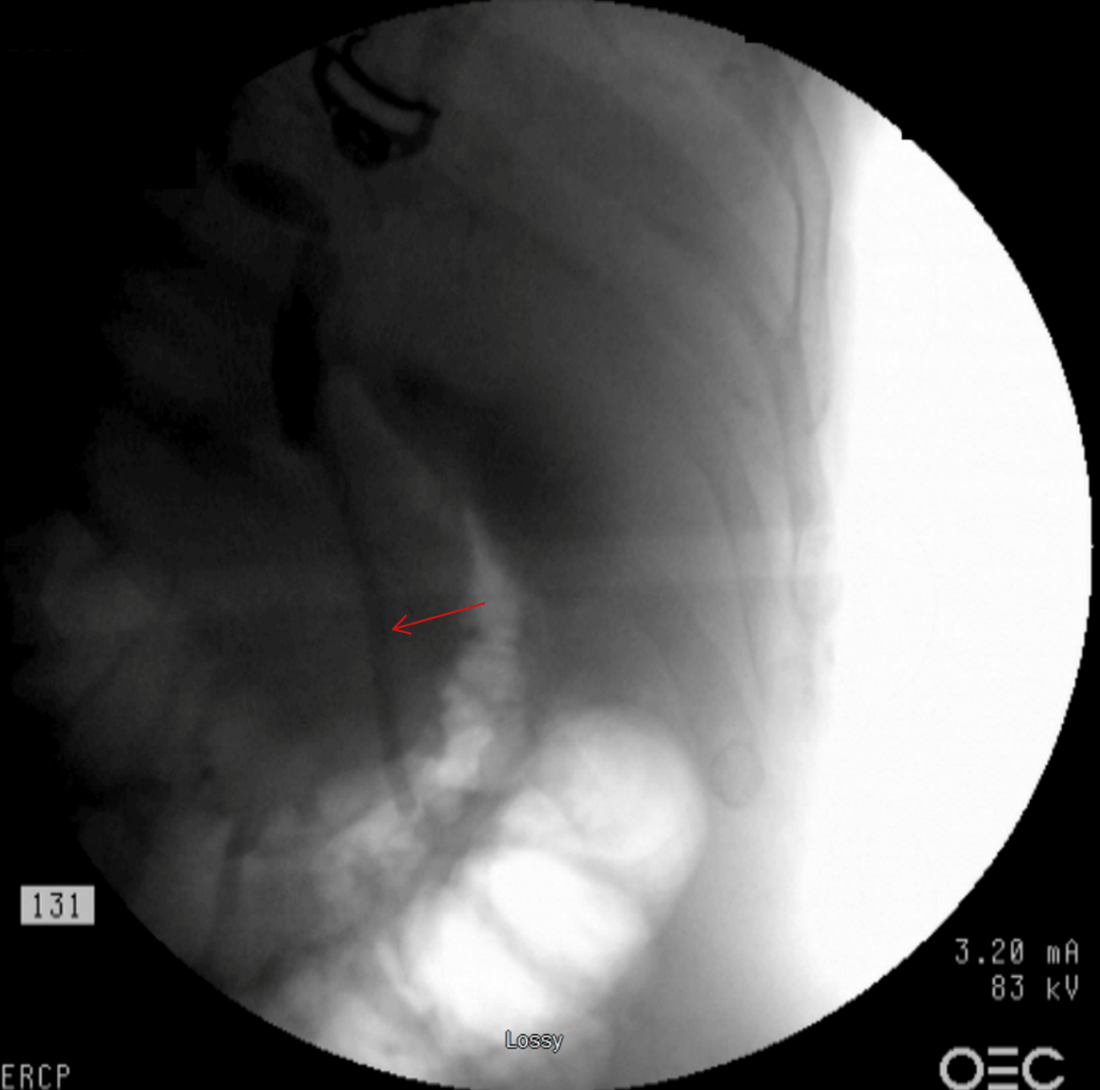
Post-endoscopic retrograde cholangiopancreatography (ERCP) demonstrating the 10 French x 9 cm plastic stent (red arrow) placed across a 4 cm long stricture in the lower common bile duct was performed.

An endoscopic retrograde cholangiopancreatography (ERCP) was performed, revealing a 4 cm long stricture in the lower CBD. Brushings of the bile duct stricture were obtained, and a 10 French × 9 cm plastic stent was placed into the CBD (Figure 2). Balloon sweeping extracted sludge but no stones. The patient did not return for scheduled follow-up stent replacement, and at a later admission, a repeat ERCP attempt was hindered by duodenal edema. The previously placed stent was partially visible in the duodenum and was removed, with no further stenting performed at that time.

**Figure 3:**
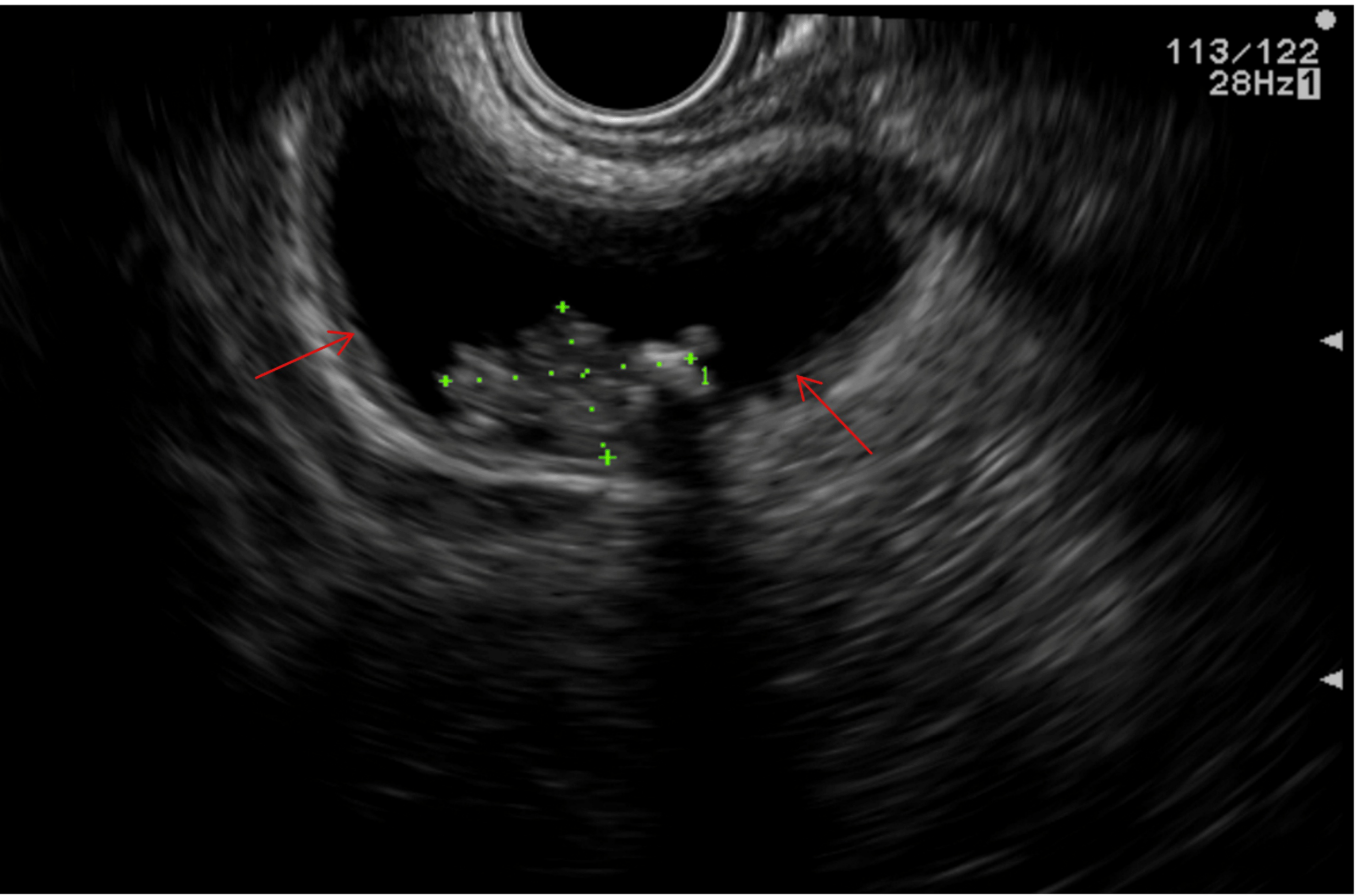
Endoscopic ultrasound (EUS) images showing a large cystic lesion in the head of the pancreas measuring 5 × 6 cm (red arrows) with features suggestive of a cystic neoplasm.

Subsequent endoscopic ultrasound (EUS) identified a large cystic lesion in the head of the pancreas measuring 5 × 6 cm with features suggestive of a cystic neoplasm (Figure 3). Fine-needle aspiration (FNA) of the cyst yielded 140 cc of turbid, brown fluid; cytology was non-diagnostic due to limited cellular material.

Over the following months, the patient experienced recurrent episodes of pancreatitis despite medical management and reported abstinence from alcohol for variable periods. Imaging studies consistently showed a mass-like fullness in the pancreatic head, a dilated pancreatic duct, and features of chronic pancreatitis with calcifications. Repeated attempts at ERCP were complicated by duodenal edema and inability to access the ampulla. A laparoscopic cholecystectomy was performed without complications.

**Figure 4:**
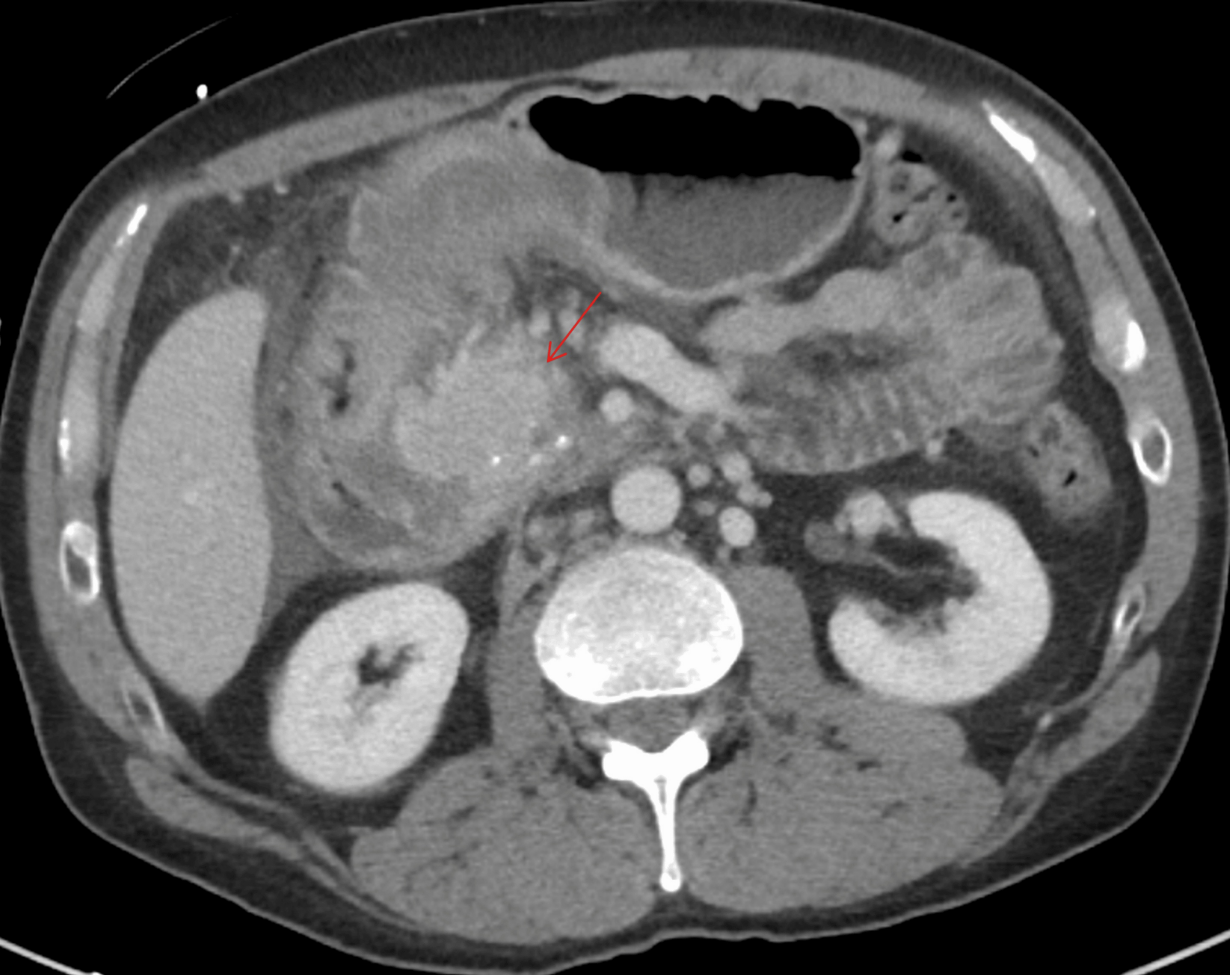
CT abdomen with IV contrast (axial view) demonstrating progression of pancreatitis with an indeterminate mass in the pancreatic head (red arrow) and associated duodenal inflammation.

At 41 years of age, the patient presented with severe epigastric pain, nausea, and vomiting. CT imaging suggested the progression of pancreatitis with an indeterminate mass in the pancreatic head and associated duodenal inflammation (Figure 4). Given concerns for neoplasm, surgical consultation was obtained, but definitive surgery was deferred due to acute inflammation and malnutrition. Nutritional optimization was initiated with nasojejunal feeding.

Despite interventions, the patient’s symptoms persisted. He developed GOO manifested by intractable nausea, vomiting, and inability to tolerate oral intake. Imaging confirmed duodenal stenosis due to a mass effect from the pancreatic head lesion. Endoscopic duodenal stenting was attempted but was complicated by stent migration and subsequent removal. Repeated episodes of electrolyte imbalances, including hyponatremia and hypokalemia, led to multiple hospital admissions. The patient also experienced seizures attributed to severe electrolyte disturbances.

At 44 years of age, the patient was admitted to the intensive care unit (ICU) with multiple electrolyte abnormalities and lactic acidosis resulting from ongoing nausea, vomiting, and poor oral intake. Diagnostic esophagogastroduodenoscopy (EGD) revealed severe gastritis without ulceration or bleeding and a near-complete obstruction of the first portion of the duodenum (D1), which could not be traversed with a wire under fluoroscopy. Given the inability to manage the obstruction endoscopically, the patient underwent an open gastrojejunostomy to bypass the obstructed duodenal segment.

Postoperatively, an upper gastrointestinal (GI) study on the first postoperative day showed that contrast flowed from the stomach through the gastrojejunostomy into the proximal small bowel without evidence of obstruction or leakage. The patient was started on a clear liquid diet, which was gradually advanced. He was discharged on postoperative day 3 with prescriptions for pancreatic enzyme supplements, a proton pump inhibitor, and metoclopramide.

## Discussion

Chronic pancreatitis is a debilitating disease characterized by persistent inflammation, fibrosis, and irreversible damage to the pancreas, often resulting from prolonged alcohol abuse [2]. In this patient, chronic alcohol consumption led to recurrent episodes of pancreatitis, the development of pancreatic calcifications, pseudocysts, and a mass-like lesion in the pancreatic head [8]. These changes contributed to biliary strictures and obstruction of the CBD, necessitating interventions like ERCP and stenting [8].

The patient developed groove pancreatitis, a rare form of segmental pancreatitis affecting the area between the pancreatic head, duodenum, and CBD [4]. This condition can mimic pancreatic carcinoma due to overlapping clinical and imaging features, making diagnosis challenging [9]. Imaging studies showed mass-like fullness in the pancreatic head and duodenal wall thickening, while EUS with FNA failed to provide a definitive diagnosis because of insufficient cellular material [7].

As the disease progressed, the patient experienced GOO resulting from duodenal stenosis caused by inflammation and fibrosis in the groove area. Symptoms included intractable nausea, vomiting, and inability to tolerate oral intake, leading to significant weight loss and malnutrition. Initial management with endoscopic duodenal stenting aimed to relieve the obstruction but was complicated by stent migration and failure to alleviate symptoms.

The patient's condition deteriorated with repeated hospital admissions for electrolyte imbalances, lactic acidosis, and episodes of seizures attributed to severe hyponatremia and hypokalemia from poor oral intake and persistent vomiting. Nutritional optimization efforts included nasojejunal feeding and total parenteral nutrition (TPN), but these were insufficient to reverse his declining nutritional status.

A significant development occurred when the patient was admitted to the ICU with multiple electrolyte abnormalities and lactic acidosis. Diagnostic EGD revealed severe gastritis without ulceration or bleeding and a near-complete obstruction of the first portion of the duodenum (D1), which could not be traversed even with a wire under fluoroscopy. This finding indicated that endoscopic interventions were unlikely to succeed in managing the obstruction.

Given the severity of the duodenal obstruction and the failure of previous endoscopic treatments, surgical intervention became necessary. The patient underwent an open gastrojejunostomy, a procedure that creates a direct connection between the stomach and the jejunum, bypassing the obstructed duodenal segment. This surgery effectively relieved the GOO, allowing for the resumption of oral intake and improvement in nutritional status.

Postoperative management included starting the patient on a clear liquid diet, which was gradually advanced as tolerated. An upper GI study on the first postoperative day confirmed that contrast flowed smoothly from the stomach through the gastrojejunostomy into the proximal small bowel without evidence of obstruction or leakage. The patient was discharged on postoperative day three with prescriptions for pancreatic enzyme supplements, a proton pump inhibitor, and metoclopramide to aid digestion, reduce gastric acidity, and enhance gastric motility, respectively.

This case demonstrates the importance of surgical intervention when medical and endoscopic treatments fail to manage complications of chronic pancreatitis effectively. Gastrojejunostomy provided a durable solution to the patient's GOO, addressing the severe symptoms that significantly impacted his quality of life. Early surgical consultation is crucial in similar cases where conservative measures are insufficient, as timely intervention can prevent further deterioration and improve outcomes.

Addressing alcohol dependence remains a critical component of comprehensive care. Continued alcohol use exacerbates pancreatic damage and undermines the effectiveness of medical and surgical treatments [10]. Collaboration with addiction specialists can provide the patient with the necessary support to achieve and maintain sobriety, which is essential for long-term disease management and prevention of recurrence [11].

In this patient, groove pancreatitis was identified as the definitive diagnosis rather than general chronic pancreatitis [12]. Distinguishing groove pancreatitis from pancreatic cancer was particularly difficult due to the overlapping clinical symptoms and imaging features [13]. The primary challenge was in making an accurate diagnosis and determining the most effective management plan. Focusing on the diagnostic difficulties and the necessity for a multidisciplinary approach provides valuable information for clinicians encountering similar cases.

In addition to describing groove pancreatitis, this case involves a complex interplay of a pancreatic cystic lesion and a bile duct stricture, both of which influenced the patient’s clinical course. The cystic lesion, initially viewed as a possible pseudocyst but later suspected to be neoplastic, prompted repeated imaging and non-diagnostic fine-needle aspirations, reflecting the challenges of differentiating among various cystic pathologies. Meanwhile, the bile duct stricture discovered on endoscopic cholangiography required stenting to alleviate obstruction and manage associated biliary symptoms. 

## Conclusions

Groove pancreatitis, a severe complication of chronic alcohol-induced pancreatitis, can result in GOO due to duodenal stenosis. When endoscopic treatments are unsuccessful or not feasible, surgical interventions like gastrojejunostomy offer effective relief by bypassing the obstructed segment. In this patient, performing an open gastrojejunostomy restored GI continuity, enabling oral intake and improving nutritional status.
